# Blue Light Promotes Neurite Outgrowth of Retinal Explants in Postnatal ChR2 Mice

**DOI:** 10.1523/ENEURO.0391-18.2019

**Published:** 2019-08-09

**Authors:** Chin-I Lin, Chuan-Chin Chiao

**Affiliations:** 1 Institute of Systems Neuroscience, National Tsing Hua University, Hsinchu 30013, Taiwan; 2 Department of Life Science, National Tsing Hua University, Hsinchu 30013, Taiwan

**Keywords:** axon regeneration, channelrhodopsin-2, intrinsic photosensitive retinal ganglion cells, light stimulation, retinal ganglion cells

## Abstract

Neurons in the adult mammalian CNS fails to regenerate after severe injury. However, it is known that an increase in neural activity occurs in mouse retinal ganglion cells (RGCs) after extrinsic stimulation and this can induce axon growth. In the present study, we applied an optogenetic approach using a mouse model, specifically involving channelrhodopsin-2 (ChR2) expression in RGCs. We investigated whether modulation of RGC neural activity exclusively by blue light stimulation is able to promote neurite outgrowth of postnatal retinal explants. The results showed that activation of RGCs expressing ChR2 by 20 Hz blue light for 1 h is a most effective way of enhancing neurite outgrowth in postnatal retinas. This is achieved via gap junctions that spread neural activity across the whole retina. Moreover, we found that activation of intrinsic photosensitive RGCs (ipRGCs) by blue light also contributes significantly to the promotion of neurite outgrowth in the same postnatal retinal explants. Our findings not only demonstrate that a short-term increase in RGC neural activity is sufficient to facilitate the neurite outgrowth of retinal explants, but also highlight the fact that the temporal pattern of neural activity in RGCs is a critical factor in regulating axon regeneration.

## Visual Abstract

**Figure:**
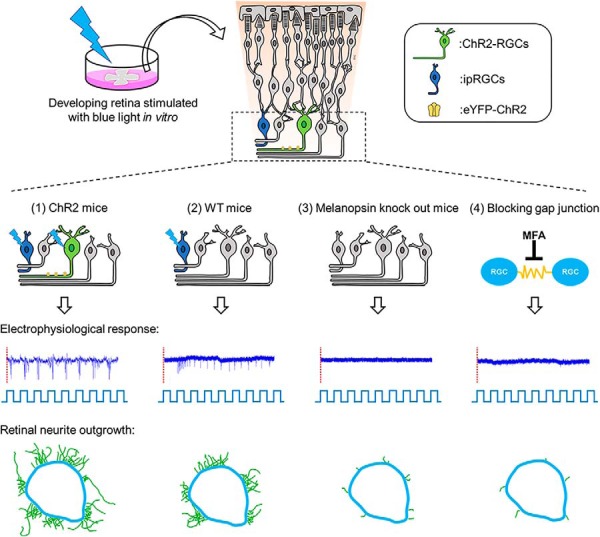

## Significance Statement

Neurons in the mammalian CNS rarely regenerate and typically die soon after injury. The use of optogenetics in promoting axon regrowth has been recognized in recent years. However, the potential of optogenetics has not been fully explored in treating optic nerve injury and glaucoma. By using the mice with channelrhodopsin-2 (ChR2) expressed specifically in retinal ganglion cells (RGCs) and stimulating the retinal explants with blue light, this project reveals that the temporal pattern of neural activity in RGCs is an important factor driving neurite outgrowth. In addition, intrinsic photosensitive RGCs (ipRGCs) also contribute in facilitating axon growth in postnatal animals. This study thus provides significant insights into the development of therapeutic strategy for axon regeneration of RGCs.

## Introduction

Retinal ganglion cells (RGCs) are the CNS neurons, the axons of which carry visual signals from the eye to various targets in the brain and they serve diverse visual functions (Masland, 2012). RGC axons rarely survive and regenerate after severe injury (Goldberg and Barres, 2000). It has been demonstrated that increased neural activity is able to promote RGC axon growth via the recruitment of neurotrophic receptors and the enhancing of gene expression (Corredor and Goldberg, 2009). For example, it has been shown that electrical stimulation is able to increase cell survival and axon regeneration in isolated RGCs (Goldberg et al., 2002; Corredor et al., 2012). It has also been reported that short-term electrical stimulation is able to enhance neurite outgrowth of retinal explants (Lee and Chiao, 2016). Furthermore, in an *in vivo* study, it was found that the enhancing of neural activity via visual stimulation, in combination with the activation of mTOR, is able to promote long-distance and target-specific regeneration of adult retinal axons (Lim et al., 2016). Taken together, these studies strongly support the idea that increased neural activity in RGCs and retinas is an effective therapeutic strategy that is likely to promote cell survival and bring about axon RGC regeneration.

Despite success applying electrical stimulation to augment neural activity and enhance axon regeneration in the retina, electrical stimulation lacks specificity and also requires direct contact with the stimulatory electrodes; these factors are likely to limit the use of this approach in future treatment. In contrast, optogenetics provides a temporally precise control of neural activity in genetically distinct cell populations that are expressing specific light-gated ion channels, one of which is channelrhodopsin-2 (ChR2; Deisseroth, 2011). Thus, light stimulation, rather than electrical stimulation, is able to allow the remote activation of specific neurons and this can be done with high temporal precision. In a recent study, it has been shown that using blue light to activate the dorsal root ganglia (DRGs) of transgenic Thy1-ChR2-YFP mice expressing ChR2 (Arenkiel et al., 2007) at 20 Hz for 1 h or 5 Hz for 4 h was able to significantly increase neurite outgrowth of DRG neurons when compared with the unstimulated controls (Park et al., 2015). In light of this promising study, the present study's aim was to investigate whether such an optogenetic approach can be applied in CNS neurons to enhance their axon growth. Specifically, postnatal retinal explants from transgenic Thy1-ChR2-YFP mice expressing ChR2 in their RGCs were activated using various temporal patterns of blue light stimulation and then their effects on neurite outgrowth were examined.

During retinal development, rods and cones do not form active synapses until approximately P10 in mice (Sernagor et al., 2001). However, postnatal retinal explants are light sensitive immediately after birth because of the early expression of intrinsic photosensitive RGCs (ipRGCs; Sekaran et al., 2005). These early mature ipRGCs serve many functions in retinal development. Like ChR2, ipRGCs are also largely sensitive to the blue spectrum of light (Berson et al., 2002), thus blue light stimulation is able to activate both ChR2-expressing RGCs and ipRGCs in postnatal retinal explants. However, the light response properties of ChR2-expressing RGCs and ipRGCs are distinctly different. The former is fast and transient (Nagel et al., 2003), and the latter is slow and long lasting (Schmidt et al., 2011). Therefore, activating postnatal retinal explants using blue light stimulation might have either a confounding or a synergistic effect on neurite outgrowth.

## Materials and Methods

### Animals

The experiments were performed on postnatal days P5 and P11 C57BL/6 (RRID: IMSR_JAX:000664), Tg (Thy1-COP4/EYFP)9Gfng (Thy1-ChR2) mice (RRID: IMSR_JAX:007615), and opn4 knock-out Cre [melanopsin knock-out (MKO); *Opn4^cre/cre^* in BL/6 background] mice (RRID: MGI:5520170; Ecker et al., 2010) of either sex. The C57BL/6 mice were obtained from the National Laboratory Animal Center in Taiwan. The ChR2 mice were originally obtained from The Jackson Laboratory in the United States, and the MKO mice were obtained from Dr. Shih-Kuo Chen at the National Taiwan University. All mice were kept in the Experimental Animal Center of National Chiao Tung University under well-controlled laboratory conditions.

### Retinal explant preparation

The mice were deeply anesthetized and killed by intraperitoneal injection of an overdose of 10 mg/kg ketamine and 10 mg/kg xylazine. To isolate the retinas, the eyeballs were enucleated using surgical scissors and bathed in warm oxygenated (95% O_2_ and 5% CO_2_) Ames’ medium (A1420; Sigma-Aldrich) containing 23 mM NaHCO_3_. Using a dissection microscope, the eyeballs were hemisected around the ora serrata with fine scissors, and the lenses were removed immediately. The retinas were then gently separated from the posterior eyecup, and the vitreous humor was removed carefully by Dumont forceps. Finally, the isolated retinas were cut into four pieces and attached ganglion cell side down onto Cell-Tak (354240; BD Biosciences) coated coverslips for retinal explant culture. All procedures were approved by the Institutional Animal Care and Use Committee of the National Tsing Hua University and were in accordance with the ARVO Statement for Use of Animals in Ophthalmic and Vision Research.

### Retinal explant culture

The retinal explants were placed in a 12-well plate and cultured in a 5% CO_2_ humidified incubator at 35°C for 5 d. Depending on the experiment, some retinal explants were stimulated using blue LED light from below for 1 h at the beginning of culture. All retinal explants were supplied daily with fresh culture medium containing Neurobasal-A (10888; GIBCO), 0.6% glucose, 1× B_27_ (GIBCO #17504-044), 1 mM sodium pyruvate (GIBCO #11360-070), 2 mM L-glutamine (GIBCO #25030081), 10 mM HEPES (GIBCO #15630-080), 100 μg/ml penicillin (GIBCO #15140-122), 2.5 μg/ml insulin (Sigma #91077), 6 mM forskolin (Sigma #F6886), and 100 nM IGF-1 (Prospec #cyt-216). In a separate experiment, 100 μM meclofenamic acid (MFA) was added in the culture medium additionally.

### Light stimulation

**Figure 1.**
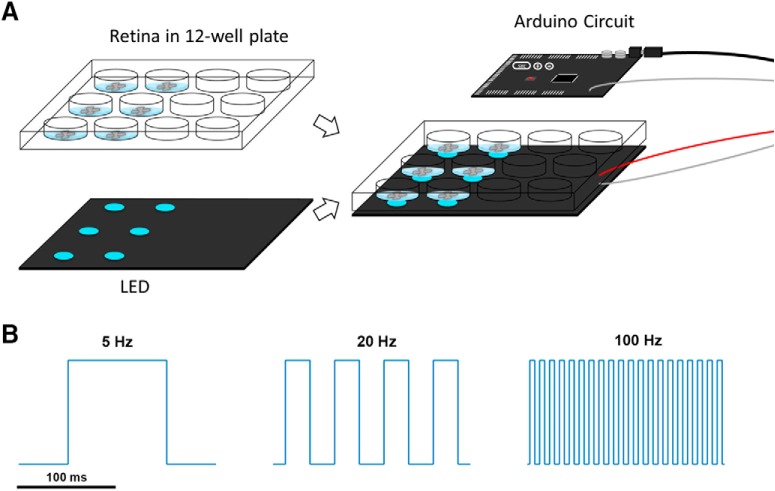
The apparatus and temporal patterns of blue light stimulation used during retinal explant culture. ***A***, The blue light LEDs (∼680 cd/m^2^, 470 nm) were powered and driven by an Arduino circuit to provide light stimulation. Retinal explants were cultured in a 12-well plate and stimulated by the LED array from below for only 1 h at the beginning of each experiment. ***B***, The temporal patterns of the 5, 20, and 100 Hz square wave light stimulations.

As part of the culture experiments, a blue light LED array (∼680 cd/m^2^, 470 nm), which was powered and driven by an Arduino microcontroller board (Arduino MEGA 2560 rev3), was used to deliver light stimulation to the retinal explants from below (Fig. 1*A*). There were three different temporal patterns of blue light used during the present study, namely, 5 Hz (100-ms pulse width), 20 Hz (25-ms pulse width), and 100 Hz (5-ms pulse width). By varying the pulse width according to the temporal frequency, the total number of photons delivered was kept the same for three different temporal patterns of light stimulation (Fig. 1*B*). To prevent the electronic circuit from being damaged in the incubator, the Arduino and the Mobile power pack were put into a separate plastic container during the experiment. To avoid light leaking from the device, a light-sealed box was used to limited the light stimulation to that required for the experiments. For the electrophysiological recordings (see below), light stimulation was provided using a stimulus generator (STG4002, Multichannel Systems), and the patterns of light pulses used were produced by a MC_Stimulus II unit (Multichannel Systems).

### Immunohistochemistry

The isolated retinas and cultured retinal explants were fixed using a mixture of 0.1% glutaraldehyde and 4% paraformaldehyde in 0.1 M PBS for 1 h at room temperature. After rinsing and cryoprotection in 30% (w/v) sucrose in dd-water, the isolated retinas were sectioned vertically using a cryostat (Leica CM3050S, Leica Biosystems) in 20-μm thickness. The whole retinas and the retinal slices were rinsed three times with PBS, and then blocked by incubation with 4% normal donkey serum and 0.1% Triton X-100 in PBS for 1 h at room temperature. The retinal explants were incubated with the primary antibody against Class III β-tubulin (TUJ1; 1:500; MMS-435P; Covance, RRID: AB_2313773) overnight at 4°C to target the outgrowth of neurites. Some of the explants were also incubated with the primary antibody against eYFP (1:100; orb256069; Biorbyt) overnight at 4°C to detect the neurites grown from ChR2-RGCs. After extensively rinsing in PBS, the secondary antibody (1:250; Alexa Fluor 488, RRID: AB_2340846, or Alexa Fluor 647, RRID: AB_2340863; Jackson) was applied overnight at 4°C to visualize the TUJ1-labeled neurites or ChR2-RGCs. In a different experiment, the isolated retinas were incubated with the primary antibody against brain-specific homeobox/POU domain protein 3A (Brn3a, C-20; 1:200; sc-31984; Santa Cruz Biotechnology, RRID: AB_2167511), caspase-3 (cleaved caspase-3, ASP175; 1:300; #9661; Cell Signaling Technology, RRID: AB_10665003), ChAT (1:100; AB114P; Millipore, RRID: AB_2313845), AMPA receptor subunit 2 (GluA2; 1:400; 182103; Synaptic Systems, RRID: AB_2113732), or vascular endothelial cell marker (CD31; 1:50; ARG52748; Arigo) overnight at 4°C to exclusively label RGCs, apoptotic cells, starburst amacrine cells, glutamate receptors, or retinal vasculatures, respectively. After extensively rinsing in PBS, the secondary antibody (1:250; Alexa Fluor 488, RRID: AB_2313584, or Alexa Fluor 647; RRID: AB_2340428, Jackson) was applied for 2 h at 25°C to visualize the targeted cells. Finally, the explants, isolated retinas, and retinal slices were mounted on slides using mounting medium containing DAPI (Vector Laboratories, RRID: AB_2336790).

### Neurite outgrowth quantification

Images of neurite outgrowth from the retinal explants were acquired using a confocal microscope (LSM510, Zeiss). Using a 20× objective, several images from different areas along the perimeter of the retinal explant were obtained. They were then stitched together to form a complete image of the retinal explant using ImageJ (National Institutes of Health, RRID: SCR_003070). The confocal images were first split into different color channels (DAPI and TUJ1), which represent, respectively, the area of the retinal explants and the area of neurite outgrowth. Only neurites that had grown out from the explants were included in the quantification and these were defined as the total neurite area. The extent of neurite outgrowth was characterized by dividing the total neurite area by the circumference of the explant. To further distinguish neurites of different lengths that had growing out from the explants, the neurite areas <100 µm, 100–200 µm, and >200 µm expanding outwards from the contour of the explants were calculated separately. Note that the neurite area <100 µm can be considered as a measure of neurite density, whereas the neurite area >200 µm represents the amount of elongated axons. A similar approach was used by Gaublomme et al. (2013) and Lee and Chiao (2016). All image analyses were performed using ImageJ.

### ChR2-RGC expression, cell apoptosis, and retinal development assessments

Images of the isolated retinas with either ChR2-RGC or caspase-3 expression and images of the retinal slices with either GluA2 or ChAT labeling were acquired using a confocal microscope (SP8, Leica). Using a 40× and 100× oil objective, several images from different retinas were obtained. To quantify the proportion of ChR2-RGCs in the retina, the numbers of Brn3a-labeled RGCs and ChR2-eYFP expressing RGCs were counted manually. To assess the extent of cell death, the caspase-3-positive cells and the DAPI-labeled cells in the retina were also calculated manually to quantify cell apoptosis. To evaluate the retinal development process, the GluA2 and ChAT expressed areas and the DAPI signal were estimated by using the ImageJ.

### Retinal angiogenesis analysis

Images of both superficial and intermediate vascular layers in the isolated retinas were acquired using a confocal microscope (LSM510, Zeiss). Using a 10× objective, several images of different areas of CD31-labeled retinal vasculatures were obtained. These images were then stitched together to form a complete image of the quarter of the whole retina using ImageJ. To quantify retinal angiogenesis, the percentage of vessel area and the density of branch points were measure using the Angio Tool Software (RRID: SCR_016393; Zudaire et al., 2011).

### Electrophysiological recording and analysis

Neural activity from the isolated retinas was recorded using an *in vitro* USB-MEA system (Multichannel Systems) with a MEA chip (60MEA200/30iR-ITO-pr-T) that consisted of 60 electrodes with diameters of 10 μm that were spaced 200 μm apart to form an 8 × 8 array. Retinas were attached to the MEA chip with the ganglion cell side down, and were constantly perfused with oxygenated Ames’ medium (1.5–2 ml/min). The temperature around the retina was kept at 37°C using a heating device (TC02, Multichannel Systems). To directly deliver the light stimulation, a blue LED was fixed below the MEA and controlled via a stimulus generator. After an on-off test (blue light with 1000-ms pulse width, 0.5 Hz) to detect neural activity in response to light, the spiking response of the retina evoked by different temporal patterns of light stimulation was recorded continuously for 1 min via 59 electrodes (one electrode serves as the ground). The interstimulus interval was 20 min to allow a full recovery of the retina's light response. In a separate experiment assessing the long-term effect of ChR2 stimulation, each retina was continuously stimulated by 5, 20, or 100 Hz blue light for 1 h during which the spiking responses were recorded for 1 min at 10-min intervals. Each retina was used for only one temporal pattern of light stimulation.

The recorded MEA datasets were first processed using MC_Rack software (Multichannel Systems, RRID: SCR_014955). The processed results were subjected to spike detection via a custom written program in MATLAB (MathWorks, RRID: SCR_001622). A high-pass filter with a cutoff frequency of 200 Hz was applied as part of the MC_Rack procedure. The spike count of each responsive MEA channel was then used to represent the retinal response to light stimulation.

### Pharmacological treatment

In the experiments where gap junction blocker was applied, oxygenated Ames’ medium containing 100 μM MFA (Reifler et al., 2015; Arroyo et al., 2016) was perfused into the MEA recording chamber. After application of the MFA for 1 h, the retina was rinsed by perfusion with normal Ames’ medium for 30 min to wash out any residual drug.

### Statistics

In the retinal explant culture experiments, the extent of neurite outgrowth under various conditions were compared using one-way ANOVA with a *post hoc* Tukey’s test. Similarly, the spiking rate of the retinas that were evoked by the different temporal patterns of blue light stimulation were compared using the same statistical process. In addition, the assessment of retinal development and angiogenesis was also conducted using the same statistics. All analyses were conducted using Excel (Microsoft, RRID: SCR_016137) and online Statistics Calculators (https://www.icalcu.com).

## Results

### Blue light stimulation promotes neurite outgrowth of retinal explants in P5 and P11 ChR2 mice

**Figure 2.**
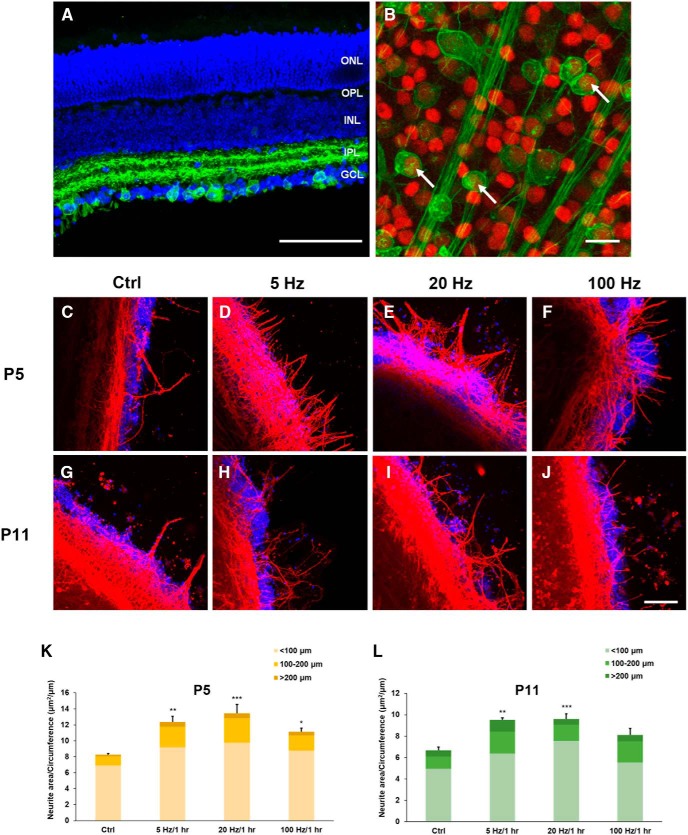
Blue light stimulation promotes neurite outgrowth of retinal explants from P5 and P11 mice with ChR2-expressed RGCs. ***A***, A vertical section through the P11 ChR2 mouse retina. DAPI was used to indicate nuclei in the retinal slice (blue), and ChR2-eYFP cells were expressed exclusively in the ganglion cell layer (GCL) with their dendrites in the inner plexiform layer (IPL). Scale bar, 100 μm. ***B***, ChR2-expressed RGCs in ChR2 mice were distributed randomly across the entire retina. Brn3a was used to label pan-RGCs (red), and ChR2-eYFP indicated ChR2-RGCs. White arrows show examples of RGCs expressing both Brn3a and ChR2. Scale bar, 20 μm. ***C–F***, Confocal images of P5 retinal explants at DIV 5 with no light stimulation (Ctrl), and with 5, 20, and 100 Hz light stimulations for 1 h at the beginning of the experiment, respectively. ***G–J***, Confocal images of P11 retinal explants at DIV 5 with the same treatments as the P5 retinal explants. All morphologically recognized neurites were TUJ1-positive (red), and DAPI was used to label the nuclei (blue). Scale bar, 100 μm. ***K***, All three light stimulation protocols promoted neurite outgrowth in the P5 retinal explants (*n* = 5 for each condition). ***L***, Similar results were obtained with the P11 retinal explants, except for the 100 Hz condition (*n* = 7 for the control; *n* = 5 for 5 Hz; *n* = 9 for 20 Hz; *n* = 6 for 100 Hz); *p < 0.05, **p < 0.01, ***p < 0.001. Error bars, mean ± SEM.

To examine whether selectively activating RGCs is able to promote neurite outgrowth of retinal explants, transgenic mice with ChR2 expressed exclusively in their RGCs were used because their activity is able to be modulated directly by blue light stimulation (Fig. 2*A*). At P11, the ChR2-eYFP expressing RGCs constituted 26.9 ± 1.3% (*n* = 7) of all the Brn3a-positive RGCs in the retina (Fig. 2*B*). Three different temporal patterns of blue light stimulation (5, 20, and 100 Hz) were applied to stimulate specifically RGCs for 1 h at the day *in vitro* (DIV) 0 using P5 and P11 retinal explants. The effect of the various different short-term RGC activations on neurite outgrowth of the P5 and P11 retinal explants was examined at DIV 5 (Fig. 2*C–L*). It was evident that all three frequencies of blue light stimulation were able to significantly enhance neurite outgrowth of P5 retinal explants when compared with the no stimulation control (*p* = 0.002 for 5 Hz, *p* < 0.001 for 20 Hz, and *p* = 0.029 for 100 Hz; Fig. 2*K*). However, the 5 and 20 Hz stimulations showed the most significant effects on the promotion of neurite outgrowth of P5 retinal explants, while the 100 Hz stimulation only had a moderate enhancement effect on neurite outgrowth. Similar results were obtained using the P11 retinal explants, where it was found that the 5 and 20 Hz stimulations were able to significantly promote neurite outgrowth compared to the no stimulation control (*p* = 0.002 for 5 Hz and *p* < 0.001 for 20 Hz; Fig. 2*L*). These results indicate that selectively increasing RGC neural activity by blue light stimulation for only 1 h at the beginning of the culture was able to effectively facilitate neurite outgrowth of both P5 and P11 retinal explants. Moreover, the findings also suggest that the temporal pattern of RGC neural activity plays an important role in determining the amount of neurite outgrowth of postnatal retinal explants.

**Figure 3.**
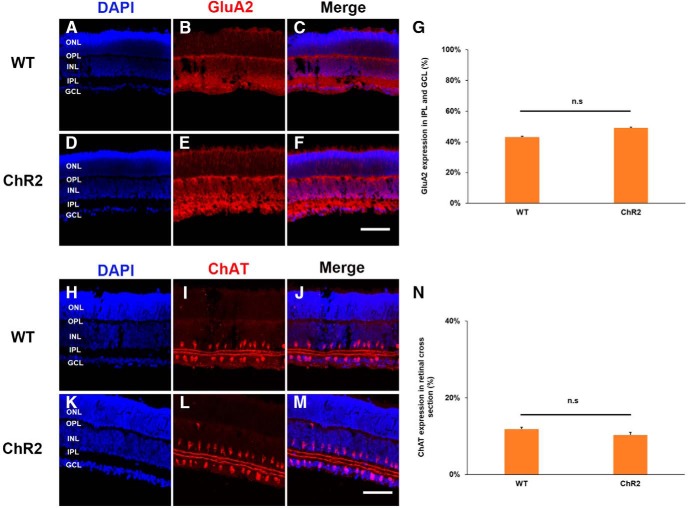
Neural developments of WT and ChR2 retinas are relatively normal in P11 mice. ***A–F***, A vertical section through P11 WT and ChR2 mouse retinas. DAPI was used to indicate nuclei in the retinal slice (blue), and GluA2 was used to label AMPA receptors (red). Scale bar, 100 μm. ***G***, GluA2 showed a similar expression level in both WT and ChR2 retinas. ***H–M***, A vertical section through P11 WT and ChR2 mouse retinas. Similarly, DAPI was used to indicate nuclei in the retinal slice (blue), and ChAT was used to label cholinergic amacrine cells (red). Scale bar, 100 μm. ***N***, ChAT also showed a similar expression level in both WT and ChR2 retinas. Taken together, these findings demonstrate that the effect of blue light stimulation on neurite outgrowth in ChR2 mice is not a result of maturation defect (*n* = 4 for WT; *n* = 5 for ChR2). n.s, *p* > 0.05. Error bars, mean ± SEM.

**Figure 4.**
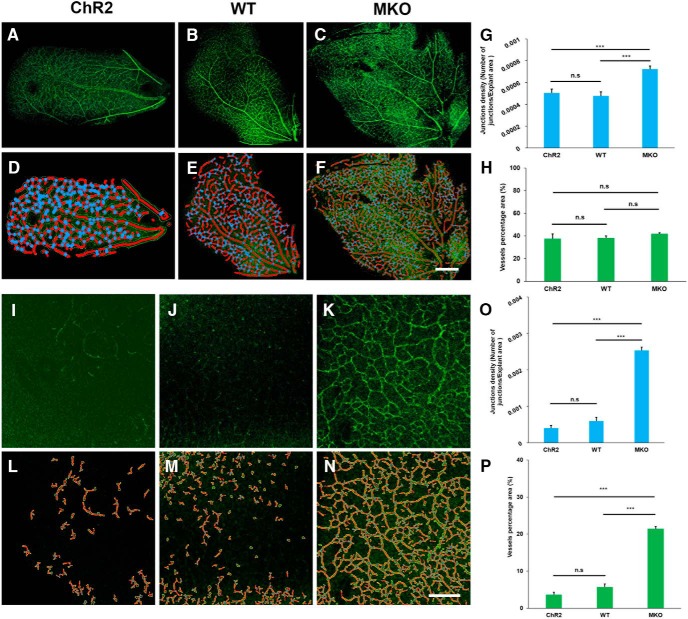
Retinal angiogenesis is accelerated in P11 MKO mice. ***A–F***, Confocal images without or with analytical labels of the superficial vascular layer of P11 retina in ChR2, WT, and MKO mice, respectively. All morphologically recognized vessels were CD31-positive, the red lines represent the skeleton of vessels, the yellow lines represent the outline of vessels, and the blue dots represent the branching points of vessels. Scale bar, 200 μm. ***G***, ***H***, Compared to ChR2 and WT mice, MKO mice showed larger junction density and vessels percentage area in the superficial vascular layer (*n* = 5 for ChR2; *n* = 11 for WT; *n* = 11for MKO). ***I–N***, Confocal images without or with analytical labels of the intermediate vascular layer of P11 retina in ChR2, WT, and MKO mice, respectively. All morphologically recognized vessels were CD31-positive. The analytical labels are the same as in panels above. Scale bar, 200 μm. ***O***, ***P***, The junction density and the percentage of vessel area significantly increased in the intermediate vascular layer of MKO mice. These results indicate that vertical angiogenic sprouting into the deeper layer of the retina occurred earlier in the absence of melanopsin (*n* = 5 for ChR2; *n* = 11 for WT; *n* = 11 for MKO). n.s, *p* > 0.05; ***p < 0.001. Error bars, mean ± SEM.

**Figure 5.**
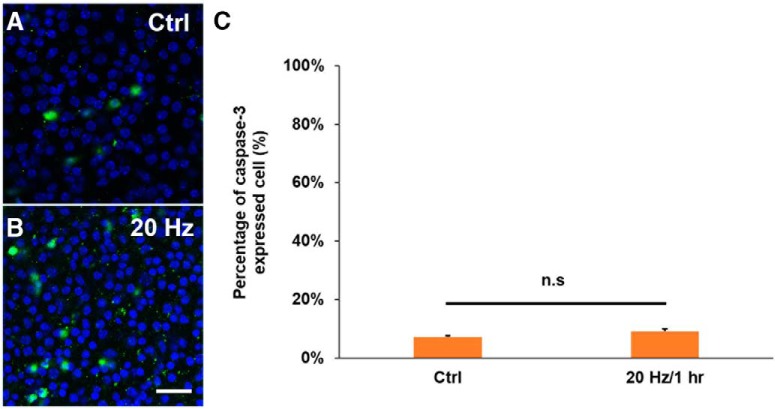
Blue light stimulation induces a low level of apoptosis in P11 ChR2 retinal explants. ***A***, ***B***, Confocal images of P11 retinal explants at DIV 5 with no light stimulation (Ctrl) and 20 Hz light stimulation for 1 h at the beginning of the experiment. DAPI was used to label cell nuclei (blue), and casepase-3 antibody was used to detect cell apoptosis (green). ***C***, Blue light stimulation did not significantly increase the expression of caspase-3 in the retinal explants (*n* = 4 for the control; *n* = 4 for 20 Hz). Scale bar, 20 μm. n.s, *p* > 0.05. Error bars, mean ± SEM.

To ensure that the developmental processes of ChR2 and wild-type (WT) mice are similar and the effect of blue light stimulation on neurite outgrowth in ChR2 mice is not a result of maturation defect, two retinal developmental markers (GluA2 and ChAT) and retinal vasculature marker (CD31) were used to examine their developmental processes from two strains of mice at P11. It was evident that neither neural development nor retinal vasculature was different in ChR2 and WT mice (Figs. 3, 4). In addition, to examine whether short-term blue light stimulation would cause significant photo-damage on retinal explants and RGCs, the retina was checked with the cell apoptosis marker caspase-3 after 20 Hz blue light exposure for 1 h. The result showed that the blue light stimulation did not significantly increase cell apoptosis when compared with the ones without blue light stimulation (Fig. 5). This result suggests that the short-term stimulation (blue light for 1 h) has a negligible photo-toxicity effect on retinal explants and RGCs.

### Activation of ipRGCs enhances the neurite outgrowth of retinal explants in P5 WT mice

**Figure 6.**
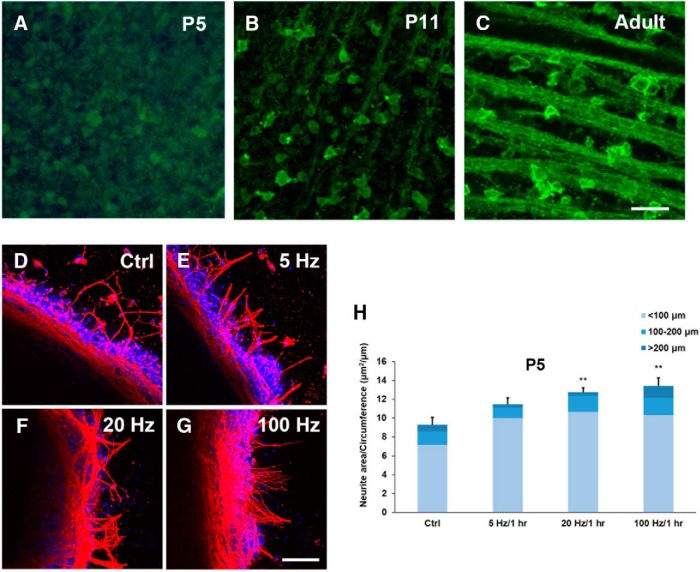
Blue light facilitates neurite outgrowth of retinal explants from P5 WT mice. ***A–C***, Confocal images of whole-mount retinas from P5, P11, and adult mice with ChR2-expressed RGCs. The fluorescence intensity indicates the eYFP-ChR2 expression level. Scale bar, 100 μm. ***D–G***, Confocal images of P5 retinal explants of WT mice at DIV 5 with no stimulation (Ctrl), and with 5, 20, and 100 Hz light stimulation for 1 h at the beginning of the experiment. Recognized neurites were TUJ1-positive (red), and DAPI was used to label the nuclei (blue). Scale bar, 100 μm. ***H***, In the absence of ChR2 expression in RGCs, the neurite outgrowth of retinal explants was still enhanced by blue light stimulation (*n* = 5 for the control; *n* = 5 for 5 Hz; *n* = 7 for 20 Hz; *n* = 5 for 100 Hz). This observation suggests that blue light was likely to activate ipRGCs within the P5 retinas at which time the retina's rods and cones have not fully developed; **p < 0.01. Error bars, mean ± SEM.

Despite the above result seems to support the hypothesis that blue light stimulation is able to effectively activate the ChR2 in the RGCs of P5 retinas, a previous study has shown that ChR2 is not expressed in RGCs until P8 (Zhang et al., 2012). We therefore decided to characterize the optogenetic expression of ChR2 in RGCs, and the eYFP-ChR2 expression level of P5, P11, and adult mice were examined (Fig. 6*A–C*). It was apparent that ChR2 is expressed prominently in a subset of RGCs at P11 and expression becomes abundant in adult; however, the expression level of ChR2 in RGCs at P5 was found to be much lower. The fact that blue light stimulation was able to effectively promote neurite outgrowth of retinal explants from P5 ChR2 mice (Fig. 2*K*) suggests that blue light must exert its effect on additional photosensitive molecules or cells in the retinal explant. Since there are no mature rod and cone photoreceptors present at P5 (Sernagor et al., 2001), the most likely candidate is the ipRGCs, which are known to be functional at a very early stage of development and to be maximally sensitive to blue light (Sekaran et al., 2005). To test the hypothesis that ipRGCs are responsible for the enhancement of neurite outgrowth on blue light stimulation in P5 ChR2 mice. Consequently, the same stimulation scheme was applied to P5 WT mouse retina and neurite outgrowth was examined at DIV 5 (Fig. 6*D–G*). The results clearly showed that 20 and 100 Hz blue light stimulation were able to significantly promote neurite outgrowth of the retinal explants from P5 WT mice, and that 5 Hz light stimulation was also able to produce a moderate effect (Fig. 6*H*). These results strongly support the hypothesis that blue light was able to act on the ipRGCs of P5 ChR2 mice to enhance neurite outgrowth of retinal explants when the rods and cones of these retinas have not fully developed and when ChR2 has not been reliably expressed in their RGCs.

### Activation of both ChR2-RGCs and ipRGCs via blue light stimulation augments neurite outgrowth of the retinal explants in P11 mice

**Figure 7.**
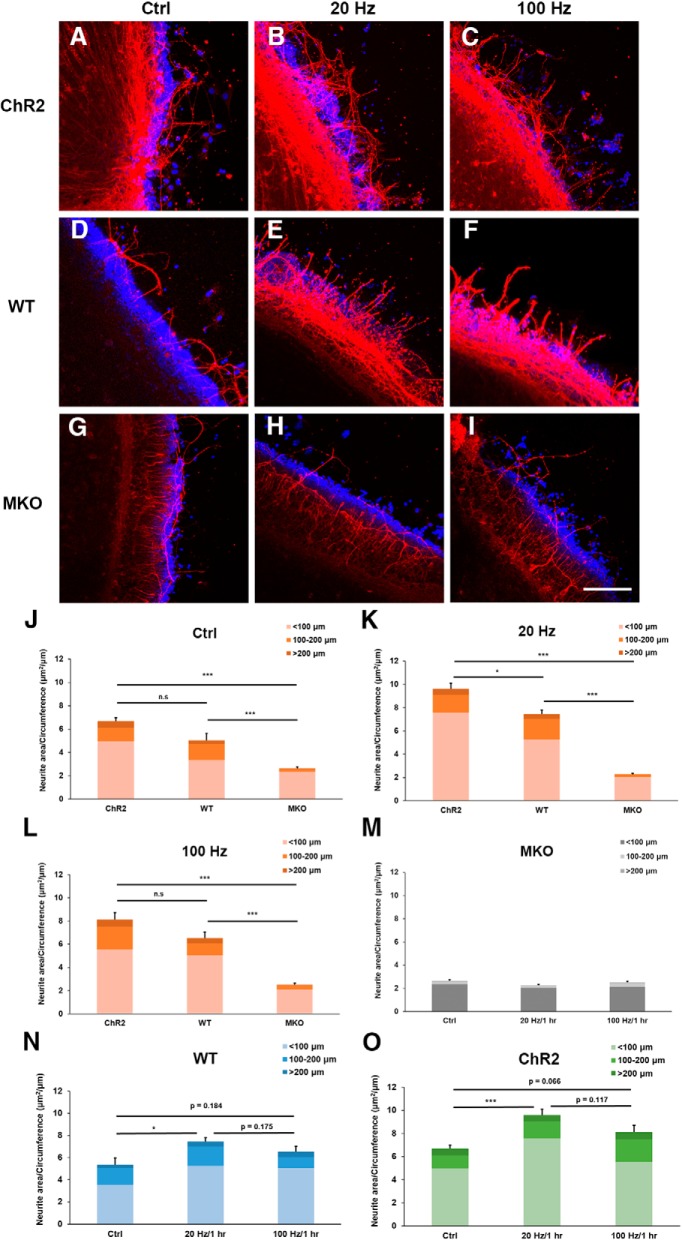
Activation of ipRGCs via blue light stimulation further enhances neurite outgrowth of retinal explants from P11 mice. ***A–C***, Confocal images of the P11 retinal explants from ChR2 mice at DIV 5 with no stimulation (Ctrl), and with 20 and 100 Hz light stimulation for 1 h at the beginning of the experiment. ***D–F***, Confocal images of P11 retinal explants from WT mice at DIV 5 with the same treatments as above. ***G–I***, Confocal images of P11 retinal explants from MKO mice at DIV 5 with the same treatments as above. Recognized neurites were TUJ1-positive (red), and DAPI was used to label the nuclei (blue). Scale bar, 100 μm. ***J***, Neurite outgrowth of P11 retinal explants was significantly different when mice with three different genetic backgrounds (ChR2, WT, and MKO) were compared, even without blue light stimulation (*n* = 7 for ChR2; *n* = 5 for WT; *n* = 6 for MKO). ***K***, Neurite outgrowth of P11 retinal explants was significantly better for ChR2 mouse retinas than for WT mouse retinas when there was 20 Hz blue light stimulation; furthermore, the neurite outgrowth was significantly reduced in MKO mice (*n* = 9 for ChR2; *n* = 5 for WT; *n* = 6 for MKO). ***L***, A similar trend was found for P11 retinal explants with 100 Hz light stimulation (*n* = 6 for ChR2; *n* = 5 for WT; *n* = 6 for MKO). ***M***, When ipRGCs and ChR2-RGCs were absent in the MKO mice, neurite outgrowth of P11 retinal explants was significantly poorer under all three conditions (no stimulation, 20, and 100 Hz blue light stimulations). ***N***, ***O***, Similar comparison schemes indicate that 20 Hz blue light stimulation enhanced neurite outgrowth more significantly than the ones without light stimulation for both WT and ChR2 mice, respectively. n.s, *p* > 0.05; *p < 0.05; ***p < 0.001. Error bars, mean ± SEM.

To further differentiate the roles of ChR2 and ipRGCs in promoting neurite outgrowth of retinal explants from P11 ChR2 mice (Fig. 2*L*), the same blue light stimulation scheme, but without 5 Hz stimulation, was applied to the retinas of three strains of P11 mice (ChR2, WT, and MKO) and their neurite outgrowth was examined at DIV 5 (Fig. 7*A–I*). At P11, rods and cones are not fully mature. However, in the ChR2 mice, with ChR2 being expressed in a subset of RGCs (Fig. 6*B*) and melanopsin being expressed in ipRGCs, these retinas are very sensitive to blue light. In the WT mice, there is only melanopsin expression in ipRGCs and no ChR2 expression in RGCs, which makes these retinas only partially sensitive to blue light. Finally, in the MKO mice, there is neither melanopsin nor ChR2 expressed in any RGCs, and thus the retina is insensitive to blue light. Interestingly, even without blue light stimulation, neurite outgrowth of the P11 retinal explants from the ChR2 and WT mice was significantly better than that in MKO mice (Fig. 7*J*). This may be the result of ambient light exposure during the dissection and handling retinal samples or some other melanopsin-dependent developing mechanisms. More importantly, under 20 Hz blue light stimulation, neurite outgrowth of the ChR2 mouse retinas was significantly greater than of the WT mouse retinas, and, conversely, neurite growth was significantly reduced when MKO mouse retinas were used (Fig. 7*K*). Similarly, under 100 Hz blue light stimulation, neurite outgrowth of the ChR2 and WT mouse retinas was also significantly greater than the MKO mouse retinas (Fig. 7*L*). If the three stimulation conditions for the MKO mouse retinas are compared, it was apparent that neurite outgrowth of the retinal explants was significantly reduced in all three cases and this effect was independent of light stimulation (Fig. 7*M*). In a similar comparison scheme, it was found that 20 Hz blue light stimulation enhanced neurite outgrowth more significantly than the ones without light stimulation for both ChR2 and WT mice (Fig. 7*N*,*O*). These findings suggest that ipRGCs play an important role in the neurite outgrowth of retinal explants at P11, and blue light is able to activate simultaneously both ChR2 and ipRGCs to further enhance neurite outgrowth in ChR2 mouse retinas.

### ChR2-expressed RGCs from P11 mice respond to blue light stimulation robustly

**Figure 8.**
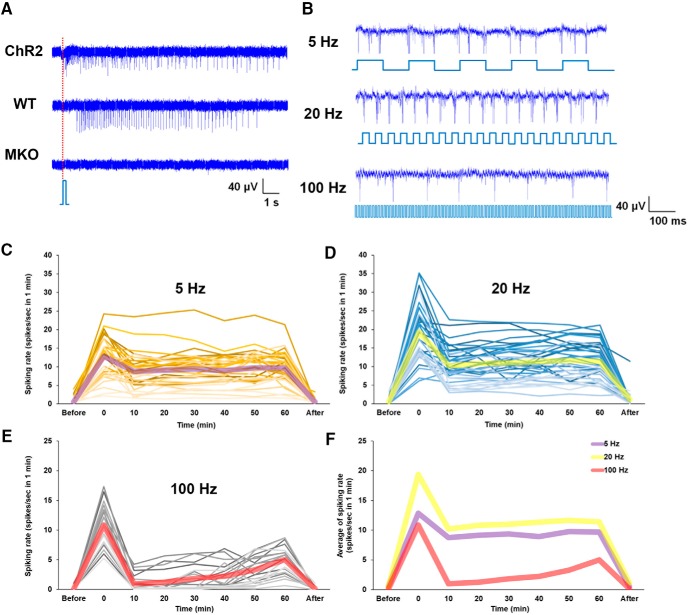
MEA recording of P11 retinas with ChR2-expressed RGCs in response to blue light stimulation. ***A***, Responses of the RGCs from the three different strains of mice (ChR2, WT, and MKO) on a 200-ms blue light stimulation. The dotted line represents the start of light stimulation. ***B***, Responses of the RGCs from ChR2 mice on 5, 20, and 100 Hz continuous blue light stimulation. When the retina was activated by 5 and 20 Hz blue light, the recorded RGC showed phase-locking responses. However, the same RGC, when stimulated by 100 Hz blue light, was not able to elicit reliable spiking responses after each light stimulation. ***C–E***, the spiking rates of the RGCs from ChR2 mouse retinas in response to 5 Hz (*n* = 35), 20 Hz (*n* = 37), and 100 Hz (*n* = 20) blue light stimulation continuously for 1 h. The spiking rate of individual RGCs at each time point was based on a 1 min measurement every 10 min. The thick semitransparent lines represent the average spiking rates. ***F***, Comparison of the average responses of the RGCs on blue light stimulation using the three temporal patterns. The RGCs responses at 5 and 20 Hz blue light were relatively sustained throughout the 1-h stimulation, while the 100 Hz blue light evoked mostly only transient responses.

To characterize the neural activity pattern of P11 retinal explants on blue light stimulation across the three strains of mice (ChR2, WT, and MKO), the responses of these RGCs to a short pulse of light (200 ms) were recorded (Fig. 8*A*). It was evident that RGCs from the ChR2 mice were activated immediately when there was blue light stimulation, and the response was long lasting, namely, for several seconds. This supports the idea that the retinas of ChR2 mice has both ChR2-expressing RGCs and ipRGCs, which are responsible for the observable immediate and delay responses, respectively. In contrast, the RGCs from WT mice showed a delayed but long-lasting response on light stimulation; this is because the retinas of WT mice have only ipRGCs at P11 that are light sensitive. Not surprisingly, MKO mice, which lack ChR2-expressed RGCs and melanopsin in ipRGCs, showed no light response at this developmental stage. To further investigate the retinal activity patterns of ChR2 mice when stimulated with the three frequencies of blue light (5, 20, and 100 Hz) at P11, RGCs from ChR2 mice were stimulated and recorded continuously for 1 h (Fig. 8*B*). We found that the spiking activities of the RGCs involved robust phase-locking responses on 5 and 20 Hz blue light stimulation. However, the same RGCs were unable to elicit such spiking responses reliably with each pulse of 100 Hz blue light stimulation. This result supports the idea that the neural activity of ChR2-expressed RGCs is able to be repeatedly evoked by light modulation at the two lower temporal frequencies (5 and 20 Hz), but not at the higher temporal frequency (100 Hz); this is consistent with the previous characterization of ChR2 kinetics (Britt et al., 2012). To quantify the neural activity of the retinal explants from the P11 ChR2 mice on stimulation with these three frequencies of blue light for 1 h, the spiking rate of individual RGCs during the first minute of every recording, which took place at 10-min intervals, was assessed (Fig. 8*C–E*). It was found that activity of all RGCs was increased immediately on light stimulation and then decreased afterward for all three frequencies. While long-term blue light stimulation inevitably reduced the responses of all RGCs, which is a sign of adaptation, the 5 and 20 Hz stimulations showed a lower effect compared to 100 Hz, and they also maintained higher plateau responses (Fig. 8*F*). These results support the hypothesis that 5 and 20 Hz blue light stimulation is able to effectively increase the neural activity of RGCs, which enhances neurite outgrowth of retinal explants. However, 100 Hz stimulation seems to be less effective at promoting neurite outgrowth of retinal explants in P11 ChR2 mice (Fig. 2*L*).

### The neural activity of ChR2-expressed RGCs evoked by blue light stimulation spreads across the retina via gap junctions in P11 mice

**Figure 9.**
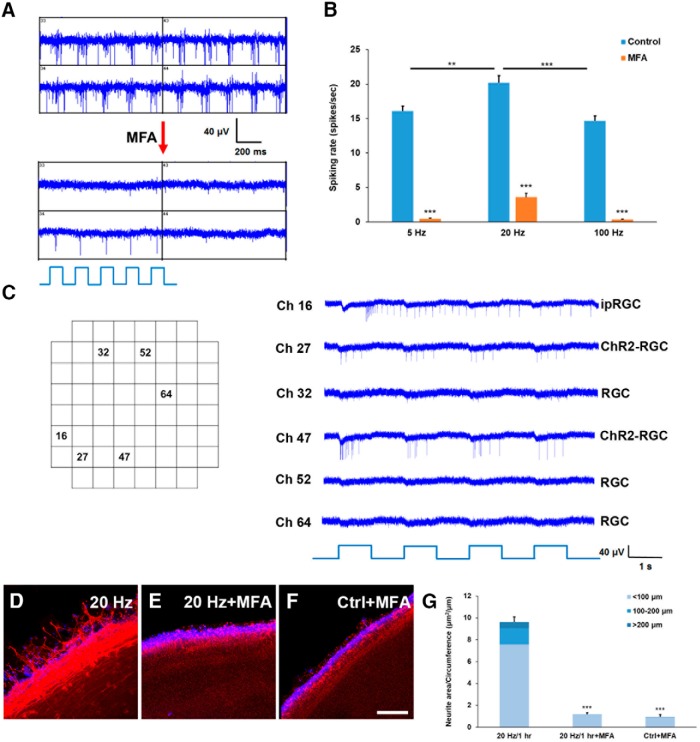
Blocking the gap junctions in the explant retinas significantly reduces the response strength and number of blue light evoked RGCs from P11 retinas of ChR2 mice. ***A***, MEA recording of four neighbor channels showing the responses of RGCs on 5 Hz blue light stimulation. All channels showed strong spiking responses before the addition of MFA (100 μM) to block the gap junctions. After blocking gap junction activity, the light responses of all channels were decreased drastically, and only one channel showed a reliable spiking response on each blue light stimulation. ***B***, Application of MFA significantly decreased the spiking rates of RGCs in response to 5, 20, and 100 Hz blue light stimulation (*n* = 52 for each condition). ***C***, An MEA recording showing a representative RGC responses on multiple 1-s blue light stimulations. After blocking the retina’s gap junctions, most channels showed no light response (only #32, #52, and #64 are shown here). However, a few channels did show a reduced yet reliable response on each blue light stimulation (and #47), and these are likely to be ChR2-expressed RGCs. Occasionally, one or two channels showed a long-lasting response to blue light stimulation, which suggests that these are one of ipRGCs present in the developing retina. ***D–F***, Confocal images of P11 ChR2 retinal explants at DIV 5 with 20 Hz light stimulation for 1 h, 20 Hz light stimulation for 1 h with 100 μM MFA, and no light stimulation with 100 μM MFA, respectively. All morphologically recognized neurites were TUJ1-positive (red), and DAPI was used to label nuclei (blue). Scale bar, 100 μm. ***G***, MFA application which blocks gap junction coupling significantly reduced neurite outgrowth of P11 ChR2 retinal explants, even under 20 Hz light stimulation for 1 h (*n* = 9 for 20 Hz; *n* = 5 for 20 Hz with MFA; *n* = 3 for the control with MFA); **p < 0.01, ***p < 0.001. Error bars, mean ± SEM.

**Figure 10.**
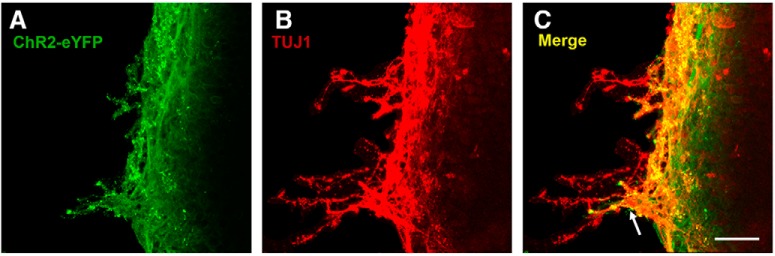
Neurites of ChR2-RGCs are observed in the outgrown neurites of the P11 ChR2 retinal explant. ***A***, eYFP antibody was used to enhance ChR2-eYFP signal from ChR2-RGCs in the retinal explant. ***B***, Recognized neurites of the retinal explant were all TUJ1-positive. ***C***, The white arrow shows the colocalization of ChR2-eYFP and TUJ1 signals indicating that some outgrown neurites were from ChR2-RGCs. Scale bar, 100 μm.

It was known that ChR2 is expressed only in a subset of RGCs in adult ChR2 mice (Thyagarajan et al., 2010), and the expression level is even lower in P11 retinas (Fig. 6*B*). Similarly, ipRGCs constitute only a small percentage of the total RGCs in the adult mouse retina (Sekaran et al., 2005). Although ipRGCs in the immature retina are more abundant than in the adult, they still make up <15% of RGCs during the developmental stages investigated here (Sekaran et al., 2005). If ChR2-expressed RGCs and ipRGCs are the only two types of cells that are light sensitive at P11 (Fig. 8*A*), given the low percentage of these cells during this developmental stage, why is blue light stimulation able to enhance the neural activity of so many RGCs in ChR2 mice (Fig. 8*C–E*)? To address this question, the gap junction blocker, 100 μM MFA, was applied to examine the effect of gap junctions on the propagation of spiking activity by ChR2-expressed RGCs and ipRGCs on blue light stimulation of the P11 retinas from ChR2 mice. We found that MFA drastically reduced the neural activity of all MEA recorded RGCs that was elicited by 5 Hz blue light stimulation, except for a few RGCs that were potentially expressing ChR2 and which still responded reliably to the light (Fig. 9*A*). By quantifying the population of RGC responses during the three different patterns of blue light stimulation (5, 20, and 100 Hz) before and after MFA application, it was found that the neural activity of the retinal explants was significantly decreased when their gap junctions were blocked (Fig. 9*B*). To visualize the light response of individual RGCs under gap junction blockade by MFA and to separate the contributions of ChR2 and melanopsin in driving the enhanced neural activity of P11 retinas on blue light stimulation, recordings from six represented MEA channels are presented in Figure 9*C*. Most channels (not shown here), including channels 32, 52, and 64, did not respond to blue light stimulation at all, and are presumably light insensitive RGCs. However, two channels (Ch 27 and 47) were responsive to the onset of light stimulation immediately and reliably and thus are likely to be ChR2-expressed RGCs. Occasionally, one or two of the 60 MEA channels, for example channel 16, showed a delayed but long-lasting spiking response on light stimulation, making them plausibly ipRGCs. These results strongly support the hypothesis that highly expressed gap junctions in the developing retina (Hansen et al., 2005) play an important role in spreading the neural activity of the relatively small percentages of ChR2-RGCs and ipRGCs present in these retinas. This results in the effective evoking of a response to blue light stimulation across the entire retina from ChR2 mice. To further confirm that the spreading and amplifying effect of the light enhanced neural activity via gap junctions was able to enhance neurite outgrowth, the medium containing MFA was used to culture P11 ChR2 retinal explants. The result was consistent with the electrophysiology experiment, in which blocking gap junction dramatically decreased neurite outgrowth of retinal explants with or without light stimulation (Fig. 9*D–G*). It was also found that some of ChR2-eYFP signals were detected in the outgrown neurites of retinal explants, though most of neurite outgrowth were from other RGCs (Fig. 10). This indicates that the blue light-sensitive RGCs spread the activity through other RGCs to promote the neurite outgrowth.

## Discussion

### Activating RGCs directly promotes neurite outgrowth of postnatal retinal explants

It has been reported that increasing the neural activity of mouse retinal explants by electrical stimulation is able to promote neurite outgrowth (Goldberg et al., 2002; Lee and Chiao, 2016). However, electrical stimulation not only activates RGCs, but also has a much broader impact on many other retinal neurons. Thus, it is difficult to attribute the effect of electrical stimulation on enhancing neurite outgrowth alone to the activation of RGCs. Using blue light stimulation to stimulate ChR2-expressed RGCs specifically allows the temporal pattern of RGC activity can be modulated directly. The present study clearly shows that activating a subset of RGCs directly, including ChR2-expressed RGCs and ipRGCs, is sufficient to promote neurite outgrowth in P5 and P11 retinal explants (Fig. 2*C–L*). This observation is consistent with previous studies wherein it has been shown that enhancement of neural activity in isolated RGCs increases their responsiveness to neurotrophic factors and promotes neurite outgrowth (Meyer-Franke et al., 1998; Goldberg et al., 2002). It also suggests that activating RGCs alone in an intact retina with specific temporal control might be an effective strategy for enhancing axon growth.

### ChR2-expressed RGCs and ipRGCs jointly contribute to the effect of blue light stimulation on promoting neurite outgrowth in P5 and P11 retinas

It is known that ChR2 expression in RGCs starts at around P8 (Zhang et al., 2012); this is because the Thy1 promoter that drives ChR2 expression only becomes active postnatally (Ting and Feng, 2013). Our confocal imaging results confirms that there is little ChR2 expression at P5, and that there is significantly more expression at P11, with even stronger expression in the adult retina (Fig. 6*A–C*). The fact that blue light stimulation is still able to significantly enhance neurite outgrowth of retinal explants in the absence of significant levels of ChR2 expression in P5 ChR2 mice (Fig. 2*K*) suggests that other light-sensitive cells must be involved in this phenomenon at this early developmental stage. Our experimental findings show that blue light stimulation is also able to promote neurite outgrowth of retinal explants even in WT mice (Fig. 6*H*), which strongly implies that ipRGCs, which are present in the retina before birth, are the light-sensitive cells (Tarttelin et al., 2003; Sekaran et al., 2005; Sexton et al., 2015). Additional experiments using MKO mice were able to show that ipRGCs are indeed activated by blue light stimulation (Fig. 7), and this supports the hypothesis that ipRGCs play a significant role in promoting axon growth during early retinal development. In the mouse retinal vasculature development, the previous study has shown that the superficial vascular plexus was formed first and the vessels sprouted to cover the entire retina at P8–P10; then the vertical sprouting occurred and the large number of vessels was found in the intermediate and deeper layers around P15 (Milde et al., 2013). To investigate whether the poor neurite outgrowth in MKO mice was associated with the development of retinal vasculature, the extent of retinal angiogenesis was examined at P11. The result indicates that MKO mice had a significantly higher level of retinal angiogenesis in both of superficial and intermediate layers (Fig. 4). This is consistent with the previous report that melanopsin regulated retinal angiogenesis and lacking melanopsin expression resulted in retinal vasculature overgrow (Rao et al., 2013). Therefore, premature retinal angiogenesis in MKO mice may have an impact on retinal neurite outgrowth as shown in the present study (Fig. 7), but the mechanism is currently unknown and is worth of investigation in the future.

At P11, both ChR2-expressed RGCs and ipRGCs are present in the retina of ChR2 mice, thus blue light stimulation is able to activate both light-sensitive cells as shown by their increased spiking rates within these RGCs. The elevated level of neural activity in the retinal explants in turn is able to promote neurite outgrowth (Fig. 2*L*). Moreover, ChR2 expressing RGCs and ipRGCs have an additive effect on the enhancement of neurite outgrowth (Fig. 7*K*), as their responses to blue light stimulation are different (Fig. 8*A*) and thus their activity patterns are able to differentially contribute to the observed effects. However, both ChR2 expressing RGCs and ipRGCs make up only a smaller percentage of the RGCs present in the retina. For example, while ∼30–40% of RGCs have been found to be ChR2-eYFP-positive in adult transgenic mice (Thyagarajan et al., 2010), only ∼20% were positive at P9 (Zhang et al., 2012) and ∼27% at P11 (Fig. 2*B*). Furthermore, the proportion of ipRGCs in the retina has been found to decrease during development, with ∼14% RGCs being ipRGCs at P0, ∼5% at P5, and only ∼3–5% in the adult retina (Sekaran et al., 2005; Ecker et al., 2010). Thus, activating these light-sensitive cells alone seems to be unlikely to produce such a significant effect on promoting the neurite outgrowth. This means that the generated neural activity needs to be amplified and spread across many more RGCs from the stimulated cells.

### Gap junctions assist the spread of the spiking responses of ChR2-RGCs and ipRGCs to other light-insensitive RGCs

It was known that gap junctions are able to assist retinal waves to propagate across the RGC layer in immature retinas and they do so by regulating RGC firing (Hansen et al., 2005). Previous studies have also shown that various types of RGCs are homologous and heterologous coupled via gap junction proteins connexin 36 and connexin 45 with each other and with amacrine cells (Bloomfield and Völgyi, 2009), and these couplings are predominant during early stages of development, including α-RGCs, γ-RGCs, direction-selective RGCs, etc. (Penn et al., 1994; DeBoer and Vaney, 2005; Chan and Chiao, 2008; Blankenship et al., 2011; Xu et al., 2013). Our results show that MFA, a gap junction blocker, is able to drastically decrease the spiking rate of all RGCs and unmask the neural response of light-sensitive ChR2-expressed RGCs and ipRGCs (Fig. 9). This suggests that the wide spread of electrical synapse activity among RGCs at this early developmental stage plays an important role in propagating the spiking activity from the light-sensitive subset of RGCs to the many other RGCs present, which in turn enhances the overall neural activity of the retinal explants (Arroyo et al., 2016). This is supported by the observation that ipRGCs formed an extensive gap junction network in the developing retina (Pérez de Sevilla Müller et al., 2010). Within this network, ipRGCs are electrically coupled to other ipRGCs and non-ipRGCs and this results in a spread of ipRGC depolarization across the whole retina. Our study demonstrates that gap junctions are not only essential for retinal wave propagation, but are also able to facilitate light-dependent axon growth within the developing retina.

### The temporal pattern of neural activity in RGCs is critical to the enhancement of neurite outgrowth of retinal explants

Using an optogenetic approach in the present study, the temporal pattern of neural activity is able to be modulated more precisely. Specifically, it was found that 5 and 20 Hz blue light stimulation is able to enhance neurite outgrowth of retinal explants significantly >100 Hz (Fig. 2*C–L*). A similar optogenetic approach was used to examine the effect of neural activity patterns on the facilitation of axon growth by dorsal root ganglia, and it was found that 20 Hz blue light stimulation for 1 h and 5 Hz blue light stimulation for 4 h resulted in the best neurite outgrowth (Park et al., 2015). This is consistent with the present results, which show that the frequency of blue light stimulation plays an important role in the regulation of the temporal pattern of RGC spiking activity and that this consequently has an effect on neurite outgrowth. It is known that applying different patterns of electrical stimulation to extrinsically modulate the neural activity of RGCs is able to bring about different effects in terms of cell survival and neurite growth (Corredor and Goldberg, 2009). This is likely to be the result of the activity by several neurotrophic factor related transcriptional pathways that are upregulated during electrical stimulation. For example, it has been reported that a 20 Hz biphasic electrical stimulation enhances IGF-1 secretion from Müller glia cells in the rat retina (Morimoto et al., 2005). In another study, electrical stimulation at a high frequency was found to increase neural activity and bring about the rapid release of a brain-derived neurotrophic factor in the dorsal root ganglia (Lever et al., 2001). It is also known that neural activity causes the release of calcium, which then regulates downstream pathways in mice that are related to cell survival and axon regeneration (West et al., 2001). Furthermore, a calcium influx is known to elevate the activity of soluble adenylyl cyclase (sAC) in isolated rat RGCs, which then promotes neurite outgrowth (Corredor et al., 2012). Taken together, it seems likely that 5 and 20 Hz blue light stimulation for 1 h are critical factors in the initiation of a cascade of cellular responses, including the release of various neurotrophic factors and the recruitment of their relevant receptors; this in turn enhances neurite outgrowth of the affected retinal explants.

The observation that blue light stimulations at 5 and 20 Hz are able to significantly increase neurite outgrowth of retinal explants from P11 ChR2 mice (Fig. 2*L*) may also be explained by the spiking responses of the ChR2-expressed RGCs on blue light stimulation, in which they show a phase-locking response under 5 and 20 Hz stimulation, while under 100 Hz stimulation was no reliable spiking response (Fig. 8*B*). This is due to the fact that ChR2, as a light-activating cation channel, and is only capable of transducing blue light into defined spike trains at frequencies up to between 40 and 50 Hz (Britt et al., 2012). Therefore, light stimulation at 5 and 20 Hz is able to elicit a relatively high spiking rate in ChR2-RGCs, while light stimulation at 100 Hz exceeded the known dynamic limit for ChR2. This result also suggests that the neural activity induced by the activation of ChR2 via blue light stimulation is positively correlated with the strength of neurite outgrowth of retinal explants. Thus, the present study supports the hypothesis that the temporal stimulation pattern, as well as magnitude of RGC neural activity, are both critical to facilitating axon regeneration.

## Synthesis

Reviewing Editor: Robert Kalb, Northwestern University Decisions are customarily a result of the Reviewing Editor and the peer reviewers coming together and discussing their recommendations until a consensus is reached. When revisions are invited, a fact-based synthesis statement explaining their decision and outlining what is needed to prepare a revision will be listed below. The following reviewer(s) agreed to reveal their identity: Anna Matynia. Note: If this manuscript was transferred from JNeurosci and a decision was made to accept the manuscript without peer review, a brief statement to this effect will instead be what is listed below.

Both reviewers found merit to the work, but there was a significant concern about the maturation state of various preparations. This issue must be resolve to make a convincing case the the effects seen are due to stimulation by blue light.

Reviewer #1 comments

eN-TCFN-0391-18X

Blue light promotes neurite outgrowth of retinal explants in postnatal ChR2 mice

Lin et al.

This manuscript details the effect of blue light on explant retinas in mice expressing channelrhodopsin-2 in retinal ganglion cells from Thy1-COP4/EYFP mice. The premise is based on enhanced outgrowth of neurites after electrical stimulation, with the hypothesis that stimulating ChR2 with blue light can provide an alternative means to more selectively provide neuronal stimulation after injury. The major concern is the appearance of the retinal explants of P11 mice.

Major points:

1. The authors show TUJ1 immunostaining of ChR2, Wt and Melanopsin knockout mice explanted at day P11, with significant differences in TUJ1 staining in the control/baseline conditions. The enhanced outgrowth post-explant could be due to a differentially developed state. Could ChR2 be altering in utero development from light exposure as light is detected in utero, and can alter vascular and neuronal development (Rao et al, Nature 2013)? Retinal wholemounts from the ChR2, Wt and MKO mice on day P5 and P11 showing retinal vasculature (e.g. Milde et al. Integr Biol 2013 as a guide) and some markers of neuronal development (examples could include VGLUT1, ribbon synapse formation, AMPA/Kainate vs nicotinic acetylcholine receptors or other appropriate markers) that indicate the developmental state of the retinas from each strain would substantiate the effect of post-explant stimulation.

2. The authors should include an image with eYFP (either endogenous or using an antibody) in order for the readers to get an idea of how many cells may be responding to the blue light stimulation that are positive for ChR2. A DIC overlay would also be helpful.

Minor points:

1. The authors use P11 retinas, which is clearly still developmental and may have no bearing on an adult retina, hence the discussion should be limited to developing retinas.

2. The authors have some poor references. The "OPN4 knock Cre (melanopsin KO, MKO)" is not properly referenced or named. Exactly which mouse are they using? The number of ipRGCs in the adult retina has been estimated as 3-5%, not the referenced 1%. Also, referencing glaucoma as a severe injury as opposed to the typical case in which there is a long-term increased IOP that causes increasing damage.

3. The authors could give background on which cells and the extent of gap junction coupling at P5 and P11 (optional but helpful).

Reviewer #2 comments

As noted above, this is a well-done paper extending prior work on the role of activity in promoting axon growth. Previous investigators have shown that electrical stimulation and visual stimulation can promote trophic responsiveness, survival and axon growth in vitro and in vivo. Here the authors take this model to the next significant advance, showing that stimulation of activity through blue light impacting onto melanopsin RGCs, and even more so onto ChR2 expressing RGCs/retinal cells, leads to increased axon growth in a reliable retinal explant model. Use of melanopsin/OPN4 knockout mouse retinas is a further strength in this paper that also advances the field, and demonstrating dependence on gap junctions to spread such activity to other cells is also a strength. There is considerable activity in the biotech/pharma world about using optogenetic approaches like these to restore vision in human patients, and this paper should add to the interest in such approaches.

There are a few minor points that would strengthen the manuscript:

1. Please include an en face retinal explant view of the ChR2 mouse retina, so the reader gets a sense of how many cells express ChR2. Some sense is derived from the MEA data in Fig 5, but it would be better to include in Fig 1 perhaps a flat mount retina showing the ChR2 expressing cells. Also, including some example cross sections to see if all the ChR2 expression is in cells in the ganglion cell layer? Or use confocal to quantify this? I think these are important because although the mice are already published, often with breeding time/generations the expression changes over time.

2. It would be helpful to include a measure of RGC survival at the end of the culture period with and without the blue light stimulation +/- ChR2 expression. It will be interesting either way to know if the light stimulation promotes survival or not.

3. Fig 4M shows the melanopsin KO cells in 3 different light stimulation conditions. Can you show the WT and ChR2 retinas in the same format, with ANOVA/t-test statistics, to understand if ChR2 allows a statistically significant increase over the WT in any of the light stimulation conditions?

4. Can you detect any ChR2 in the neurites growing from the explants? Again it would be interesting either way to know if most of the axons are Chr2 positive. This could also be said for OPN4, perhaps requiring an OPN4-GFP mouse...

5. Reference is made in the discussion but I cannot find data in the results/figures--does MFA blockade of gap junctions block neurite outgrowth? Where is the quantification of this?

## Author Response

Reviewer #1 comments

This manuscript details the effect of blue light on explant retinas in mice expressing channelrhodopsin-2 in retinal ganglion cells from Thy1-COP4/EYFP mice. The premise is based on enhanced outgrowth of neurites after electrical stimulation, with the hypothesis that stimulating ChR2 with blue light can provide an alternative means to more selectively provide neuronal stimulation after injury. The major concern is the appearance of the retinal explants of P11 mice.

Reply: We have carefully characterized and compared the developmental process of ChR2 and wild type P11 mice. We provided substantial evidence in the revised manuscript to demonstrate that the appearance of the retinal explants of P11 mice is relatively normal (Fig. 8).

Major points:

1. The authors show TUJ1 immunostaining of ChR2, Wt and Melanopsin knockout mice explanted at day P11, with significant differences in TUJ1 staining in the control/baseline conditions. The enhanced outgrowth post-explant could be due to a differentially developed state. Could ChR2 be altering in utero development from light exposure as light is detected in utero, and can alter vascular and neuronal development (Rao et al, Nature 2013)? Retinal wholemounts from the ChR2, Wt and MKO mice on day P5 and P11 showing retinal vasculature (e.g. Milde et al. Integr Biol 2013 as a guide) and some markers of neuronal development (examples could include VGLUT1, ribbon synapse formation, AMPA/Kainate vs nicotinic acetylcholine receptors or other appropriate markers) that indicate the developmental state of the retinas from each strain would substantiate the effect of post-explant stimulation.

Reply: Although light can be detected in utero, and can alter vascular and neuronal development, ChR2 is not expressed until P8 in mice (see Fig. 3A in the present study). Thus, eye development of ChR2 mice is unlikely to be affected in utero. We have used antibodies against CD31 (label retinal vasculature), GluA2 (label AMPA receptors), and ChAT (label starburst amacrine cells) to target specific cells in wholemounts and retinal slices of ChR2, Wt and MKO mice on day P11. The developmental states of ChR2 and Wt mice are comparable (Fig. 8), while the retinas of MKO mice show an earlier developmental state (Fig. 9), which is consistent with the result reported in the previous study (Rao et al, Nature 2013). We have included these results in the revised manuscript as Figure 8 and Figure 9 to substantiate the effect of post-explant stimulation.

2. The authors should include an image with eYFP (either endogenous or using an antibody) in order for the readers to get an idea of how many cells may be responding to the blue light stimulation that are positive for ChR2. A DIC overlay would also be helpful.

Reply: We have used a RGC-specific marker Brn3a to investigate what percentage of ChR2-RGCs was co-localized with RGCs in the retina of P11 ChR2 mice. We found that ChR2-eYFP expressing RGCs constituted 26.9 {plus minus} 1.3% of all the Brn3a positive RGCs in P11 ChR2 mice. This result is consistent with previous studies, in which approximately 30-40% of RGCs have been found to be ChR2-eYFP positive in adult ChR2 mice (Thyagarajan et al., 2010) and only about 20% were positive at P9 (Zhang et al., 2012). We have included this result in the revised manuscript as Figure 2B and reported the percentage in the Results section.

Minor points:

1. The authors use P11 retinas, which is clearly still developmental and may have no bearing on an adult retina, hence the discussion should be limited to developing retinas.

Reply: We have limited the interpretation and discussion of these results to developing retinas. The speculation of the effect on adult retinas has been removed.

2. The authors have some poor references. The “OPN4 knock Cre (melanopsin KO, MKO)” is not properly referenced or named. Exactly which mouse are they using? The number of ipRGCs in the adult retina has been estimated as 3-5%, not the referenced 1%. Also, referencing glaucoma as a severe injury as opposed to the typical case in which there is a long-term increased IOP that causes increasing damage.

Reply: We have properly referenced the MKO mouse line used in the present study (Ecker et al., 2010), and we have also added the detailed animal info in the Methods section. Indeed, the number of ipRGCs in the adult retina has been estimated as 3-5%, and we have cited the correct reference (Ecker et al., 2010) in the revised manuscript. We have also removed the example of glaucoma in the Introduction section.

3. The authors could give background on which cells and the extent of gap junction coupling at P5 and P11 (optional but helpful).

Reply: Indeed, previous studies have shown that various types of RGCs are homologous and heterologous coupled via gap junction proteins connexin 36 and connexin 45 with each other and with amacrine cells (Bloomfield and Völgyi, 2009 ). We have added the information about the cell types and the extent of gap junction coupling during early stages of retinal development in the Discussion section. This includes α-RGCs, γ-RGCs, and direction-selective RGCs, etc. (Penn et al., 1994, DeBoer and Vaney, 2005; Blankenship et al., 2011; Chan and Chiao, 2008; Xu et al., 2013). Coupled ipRGCs are also found at developing stages, which is known to regulate retinal waves (Arroyo et al., 2016).

Reviewer #2 comments

As noted above, this is a well-done paper extending prior work on the role of activity in promoting axon growth. Previous investigators have shown that electrical stimulation and visual stimulation can promote trophic responsiveness, survival and axon growth in vitro and in vivo. Here the authors take this model to the next significant advance, showing that stimulation of activity through blue light impacting onto melanopsin RGCs, and even more so onto ChR2 expressing RGCs/retinal cells, leads to increased axon growth in a reliable retinal explant model. Use of melanopsin/OPN4 knockout mouse retinas is a further strength in this paper that also advances the field, and demonstrating dependence on gap junctions to spread such activity to other cells is also a strength. There is considerable activity in the biotech/pharma world about using optogenetic approaches like these to restore vision in human patients, and this paper should add to the interest in such approaches.

Reply: We thank that the reviewer gave a nice summary of our work and appreciated our effort in this research direction.

There are a few minor points that would strengthen the manuscript:

1. Please include an en face retinal explant view of the ChR2 mouse retina, so the reader gets a sense of how many cells express ChR2. Some sense is derived from the MEA data in Fig 5, but it would be better to include in Fig 1 perhaps a flat mount retina showing the ChR2 expressing cells. Also, including some example cross sections to see if all the ChR2 expression is in cells in the ganglion cell layer? Or use confocal to quantify this? I think these are important because although the mice are already published, often with breeding time/generations the expression changes over time.

Reply: We have included an en face image of the P11 ChR2 mouse retina (Fig. 2B) showing that the ChR2-eYFP expressing RGCs constituted 26.9 {plus minus} 1.3% of all the Brn3a positive RGCs (a pan RGCs marker). In addition, we have also shown a cross section image of ChR2 retina (Fig. 2A) indicating that ChR2 is expressed exclusively in the ganglion cell layer (GCL) with their dendrites distributed in the inner plexiform layer (IPL).

2. It would be helpful to include a measure of RGC survival at the end of the culture period with and without the blue light stimulation +/- ChR2 expression. It will be interesting either way to know if the light stimulation promotes survival or not.

Reply: We have done immunostaining using an antibody against caspase-3 as a cell apoptosis marker. The result showed that the blue light stimulation did not significantly increase cell apoptosis when compared with the ones without blue light stimulation. This suggests that the short-term stimulation (blue light was only applied for one hour) has much less photochemical damage on retinal explants than the long-term stimulation condition. We have included the data in the revised manuscript as Figure 10, and noted it in the Discussion section.

3. Fig 4M shows the melanopsin KO cells in 3 different light stimulation conditions. Can you show the WT and ChR2 retinas in the same format, with ANOVA/t-test statistics, to understand if ChR2 allows a statistically significant increase over the WT in any of the light stimulation conditions?

Reply: We have added the same format of comparison like Fig. 4M in the revised manuscript as Fig. 4N and Fig. 4O, and did a similar statistical analysis to compare the light effect. Indeed, it was found that 20 Hz blue light stimulation enhanced neurite outgrowth more significantly than the ones without light stimulation for both ChR2 and wild type mice. We have added this info in the Results section.

4. Can you detect any ChR2 in the neurites growing from the explants? Again it would be interesting either way to know if most of the axons are Chr2 positive. This could also be said for OPN4, perhaps requiring an OPN4-GFP mouse...

Reply: We have used eYFP antibody to amplify ChR2-eYFP signals from the outgrown neurites of retinal explants in ChR2 mice, and we found that some of ChR2-eYFP signals were detected in the outgrown neurites of retinal explants, though most of neurite outgrowth were from other RGCs (Fig. 7). This result indicates that the blue light sensitive RGCs spread the activity through other RGCs to promote the neurite outgrowth. We have added this info in the Results section. However, we did not use the OPN4-GFP mouse to do the similar experiment. The main reason is that our estimation of ChR2-RGCs in the RGC population is about 27% at P11, but the percentage of ipRGCs is estimated less than 5% at the same developmental stage, thus it would be much difficult to find the neurites growing from the explants are originated from ipRGCs.

5. Reference is made in the discussion but I cannot find data in the results/figures--does MFA blockade of gap junctions block neurite outgrowth? Where is the quantification of this?

Reply: Indeed, we did not do the experiment that the reviewer requested in the original manuscript. In the revised manuscript, we have added this line of results in new Figure 6 (Panels D, E, F, and G). In this experiment, the medium containing MFA was used to culture P11 ChR2 retinal explants. The result was consistent with the electrophysiology experiment, in which blocking gap junction dramatically decreased neurite outgrowth of retinal explants with or without light stimulation (Fig. 6D-6G).
